# Establishment and Application of Rapid Diagnosis for Reverse Transcription-Quantitative PCR of Newly Emerging Goose-Origin Nephrotic Astrovirus in China

**DOI:** 10.1128/mSphere.00380-18

**Published:** 2018-11-07

**Authors:** Xiaoyuan Yuan, Kai Meng, Yuxia Zhang, Lihong Qi, Wu Ai, Youling Wang

**Affiliations:** a Institute of Poultry Research, Shandong Academy of Agricultural Sciences, Ji'nan, Shandong, People's Republic of China; Icahn School of Medicine at Mount Sinai

**Keywords:** goose-origin astrovirus, RT-qPCR, TaqMan probe

## Abstract

In 2017, a new type of goose-origin astrovirus (GoAstV) that is completely different from previously identified avian astroviruses (which have only 30.0% to 50.5% homology with GoAstV) has been isolated from diseased geese in China. This disease can cause joint swelling in sick geese, and the anatomy shows a clear precipitation of urate in the kidney. The rate of death and culling can reach more than 30%, revealing the disease’s severe pathogenicity. To quickly and accurately diagnose the newly emerging disease, we established a highly specific reverse transcription-quantitative PCR (RT-qPCR) method of detecting GoAstV. Sensitivity testing showed that the minimum amount of test sample for this method is 52.5 copies/μl. Clinical application confirmed that this method can quickly and effectively detect GoAstV, providing a diagnostic platform for the prevention and control of goose disease.

**IMPORTANCE** Goose-origin astrovirus (GoAstV), as a newly emerging virus in 2017, is different from previously known astroviruses in the genus Avastrovirus. So far, few studies have focused on the novel virus. Considering the infectious development of astrovirus (AstV), we established a reverse transcription-quantitative PCR (RT-qPCR) assay with a strong specificity to quickly and accurately diagnose GoAstV. Confirmed by clinical application, this method can quickly and accurately detect prevalent GoAstV. The assay is thus convenient for clinical operation and is applicable to the monitoring of GoAstV disease.

## Abstract (precis)

Goose-origin astrovirus (GoAstV), as a newly emerging virus in 2017, is different from previously known astroviruses in the genus Avastrovirus. So far, few studies have focused on the novel virus. Considering the infectious development of astrovirus (AstV), we established a reverse transcription-quantitative PCR (RT-qPCR) assay with a strong specificity to quickly and accurately diagnose GoAstV. Confirmed by clinical application, this method can quickly and accurately detect prevalent GoAstV. The assay is thus convenient for clinical operation and is applicable to the monitoring of GoAstV disease.

## INTRODUCTION

Astroviruses, of the family Astroviridae, are single-stranded, positive-sense RNA viruses with genome lengths of 6.4 to 7.9 kb (1). Astroviridae species can be divided into mammalian and avian astroviruses, according to the different types of infected hosts. Mammalian astroviruses are further divided into human, cat, swine, sheep, and mink astroviruses, depending on their hosts. Avian astroviruses include duck astrovirus, turkey astrovirus, avian nephritis virus, and chicken astrovirus (2–4).

In the second half of 2017, a new type of goose-origin astrovirus (GoAstV) has been isolated from diseased geese in China (5, 6). In our early study, this disease can cause joint swelling and a clear precipitation of urate in the kidney. Phylogenetic analysis of partial open reading frame (ORF) 1b protein revealed that the new GoAstV differed from previously reported avian AstVs in terms of genotypes, and its homology with other avian AstVs was only 30% to 50.5%. Current methods for viral isolation and identification are unsuitable for clinical application because of their time-consuming and labor-intensive processes. Moreover, GoAstV has no hemagglutination, does not grow on chicken or duck embryos, and only reproduces on goose embryos. At present, ordinary reverse transcription-PCR (RT-PCR) is commonly used to detect GoAstV. However, this method can easily cause cross-contamination between specimens, requiring gel electrophoresis, and it is unsuitable for detection in a large number of clinical samples (7). Considering the genetic diversity and possible interspecies transmissions of GoAstV, we used reverse transcription-quantitative PCR (RT-qPCR) to establish a rapid diagnosis of the virus (8). According to document retrieval, this study is the first to develop a RT-qPCR diagnosis for the novel GoAstV.

## RESULTS

**FIG. 1:**
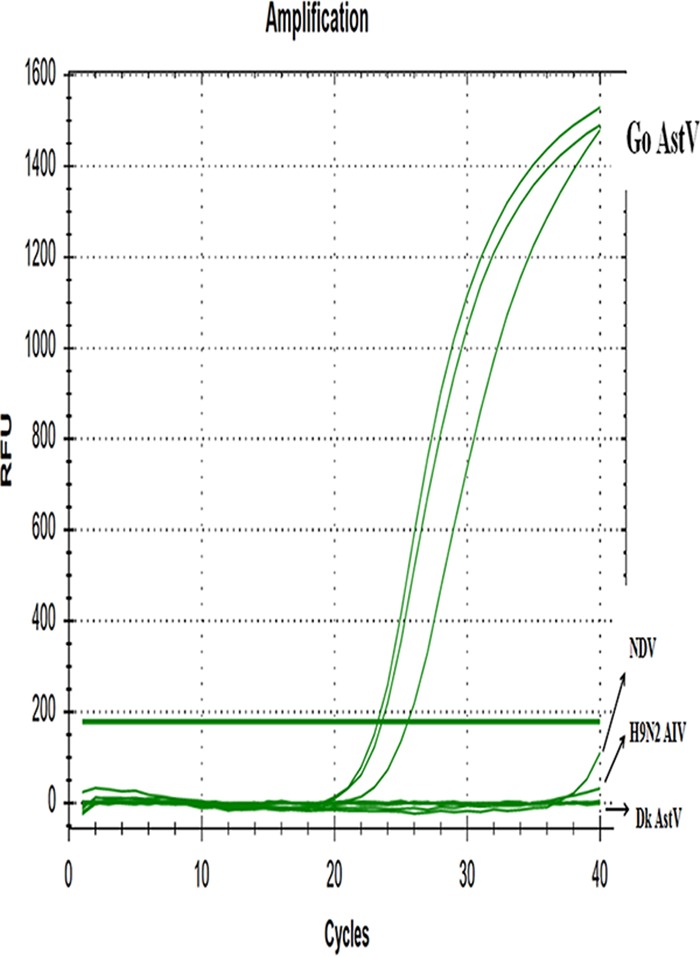
RT-qPCR amplification of GoAstV. Only GoAstV showed a positive ﬂuorescence signal, and no positive signal was observed with Newcastle disease virus (NDV), H9N2 subtype AIV, and duck (DK) AstV.

The optimal reaction conditions for quantitative PCR (qPCR) were as follows: 95°C for 2 min, followed by 40 cycles of 94°C for 5 s and 60°C for 30 s. The fluorescence signal was collected at 60°C. After qPCR reaction, three specific amplification curves (SD18 triplicate samples) appeared in the reaction channel, and the threshold cycle (*C_T_*) values were 20.1 to 20.6 (Fig. 1). Thus, the sample was judged as positive. No obvious amplifications were showed in the controls (Fig. 1).

**FIG. 2:**
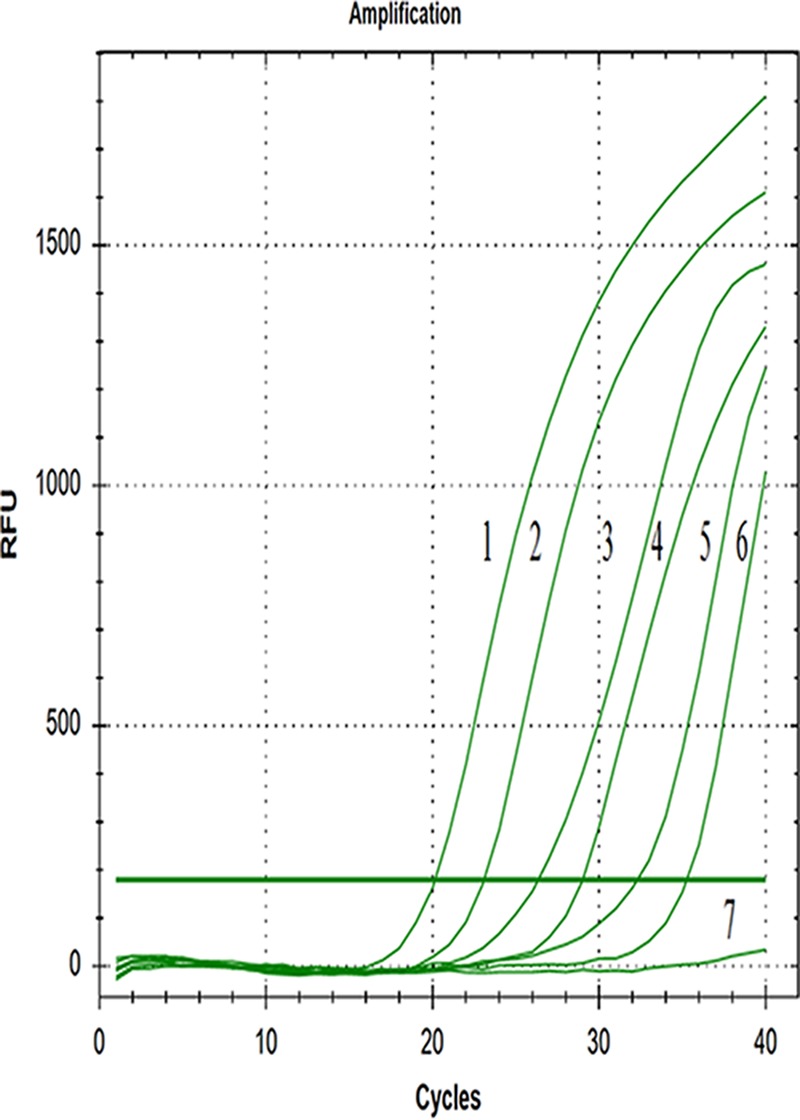
Sensitivity test for RT-qPCR assay of GoAstV. The lowest copy number that could be determined was up to 52.5 copies/μl (lane 6). Lanes 1 to 6 were the templates, with concentrations ranging from 5.25 × 10^6^ to 5.25 × 10^1^ copies/μl; lane 7, with 5.25 copies/μl, had no positive signal.

**FIG. 3:**
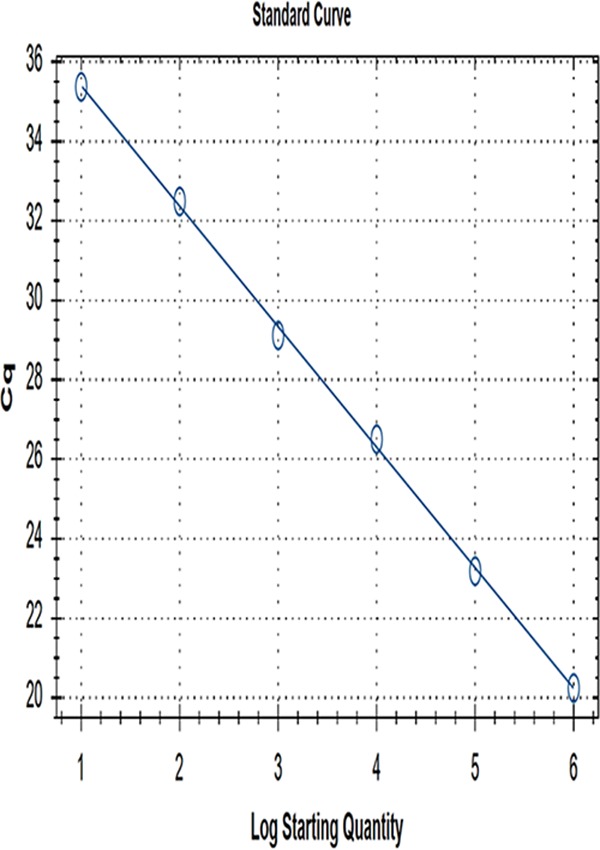
Standard curve for RT-qPCR assay of GoAstV. Cycle values (*C_q_*) (*y* axis) are plotted versus common logarithmic concentrations of plasmid copies (*x* axis), where *y* = −3.033 × log *x* + 38.438.

In sensitivity tests of qPCR, the template could still be effectively amplified when its content was 52.5 copies/μl and could not be amplified when it was 5.25 copies/μl (Fig. 2). For the standard curve established by GoAstV, the correlation coefficient (*R*^2^) was 0.999, the slope was −3.033, and the intercept was 38.438. Thus, a linear relation was obtained between copy number (*x* axis) and quantification cycle (*C_q_*) value (*y* axis), as follows: *y* = −3.033 × log *x* + 38.438 (Fig. 3).

Five clinically suspected astrovirus-infected goose tissue samples were confirmed positive via the above established method. Positive samples were verified as GoAstV by later sequence determination, whereas only four positive samples were verified by RT-PCR test. For the 40 goose embryo-inoculated virus fluids of SD18, the positive rates of RT-qPCR and RT-PCR were 90% (36/40) and 82.5% (33/40), respectively. The clinical application test results are shown in Table 1.

**TABLE 1:** Clinical detection of RT-qPCR

Sample	Result of^*a*^:		Sequence determination
	RT-qPCR	RT-PCR	
SD18	P	P	P
SD182	P	N	P
JN1	N	N	
GD	P	P	P
YU	P	P	P
Xu17	N	N	
Xu18	N	N	
SD17	P	P	P
JN2	N	N	
Inoculated embryos	36P/4N	33P/7N	

a P, positive; N, negative.

## DISCUSSION

Given that GoAstV disease is a new outbreak, few studies have focused on this novel GoAstV. In early studies, this disease can cause over 30% mortality rate and rare urate nephritis. Genetic evolution analysis of ORF 1b revealed that GoAstV shared only 30.0% to 50.5% homology with other avian AstVs (9–11).

In terms of the rapid development of AstVs and genetic diversity (5, 12–14), the simple RT-qPCR detection technology established in this paper can rapidly, specifically, and quantitatively measure the newly generated GoAstV. The proposed method has higher sensitivity with a smaller sample than the traditional detection methods. It is thus suitable for clinical application in the laboratory.

## MATERIALS AND METHODS

### Strain and reagents.

The GoAstV representative isolate SD18, goose-origin Newcastle disease virus (NDV), goose-origin H9N2 subtype AIV, and duck AstV used in the experiment were isolated and identified by the SPF Chicken Research Center of Shandong (China). GoAstV393 was a positive plasmid for a 1b protein fragment cloned into PMD18-T (5, 15). The viral simple RNA kit was from BioEr, Inc., and the qPCR kit and qPCR diluent were from TaKaRa Bio, Inc.

### Design of probe and primers.

On the basis of the conserved ORF 1b protein sequence-encoded RNA-dependent RNA polymerase (RdRP) of GoAstV from GenBank, Primer ExPress 3.0 software was used to design amplification primers and TaqMan probe; the melting temperature (*T_m_*) verification analysis was performed; and hairpins, self-dimers and cross-dimers in primer pairs were avoided. The designed primers and probe were evaluated with BLASTn (BLAST, https://www.ncbi.nlm.nih.gov/blast/). All primers were synthesized by BGI (China). The forward primer was GoAstV-F (5′-TGGTGGTGGTGCGGTTTT-3′ [nucleotide positions 14 to 31]), the reverse primer was GoAstV-R (5′-GGGCAACGTACCATCATAACG-3′ [nucleotide positions 46 to 66]), and the TaqMan probe was GoAstV-Probe (5′-FAM-TGTAGAGACGGACTGGAC-MGB-3′ [nucleotide positions 27 to 44], where FAM is 6-carboxyfluorescein and MGB is the minor groove binder).

### RT-qPCR amplification.

The viral RNA was extracted by the simple RNA kit, in accordance with the manufacturer’s protocol. The extracted RNA was used immediately. In the inverse transcription system, 4 μl of 5× PrimeScript buffer (for qPCR), 1 μl of PrimeScript reverse transcriptase (RT) enzyme mix, 0.5 μl of random primer (50 μM), 1 μg of RNA template, and RNase-free H_2_O were supplemented to 20 μl. Reaction conditions were as follows: 42°C for 15 min, 85°C for 2 min, and ending at 4°C.

The qPCR was conducted in a CFX-96 system (Bio-Rad Inc., USA). An optimal qPCR system contained the following: 10 μl of 2× Premix Ex (for qPCR), 0.2 μl of forward primer (10 μM), 0.2 μl of reverse primer (10 μM), 0.5 μl of probe (10 μM), 2 μl of cDNA, and 7.1 μl of nuclease-free H_2_O.The optimal reaction temperature of the primers was tested from 55°C to 65°C, with a temperature gradient of 2°C. The sample was set up in triplicates, and goose-origin NDV, goose-origin H9N2 subtype AIV, and duck AstV were set as controls. The controls were diluted in known negative samples to verify the presence of inhibitors in the sample.

### Sensitivity tests and standard curve.

The concentration of the GoAstV393 plasmid, 5.25 × 10^6^ copies/μl, was determined using a NanoDrop microspectrometer. Sensitivity tests were performed using a 10-fold dilution series of the extracted plasmid. The positive plasmid AstV393 was 10-fold serially diluted to 5.25 copies/μl by special diluents for standard curve. Using double-distilled (ddH_2_O) as the negative control, we performed sensitivity tests under the optimized reaction conditions. Meanwhile, the standard curve of qPCR for GoAstV was established.

### Clinical detection of RT-qPCR.

Nine clinically suspected astrovirus-infected goose tissue samples were collected from 2017 to 2018. All samples were liver tissue, except for sample JN2, from spleen. Samples were processed by ultrasonic comminution, and supernatants were taken for RT-qPCR after centrifugation.

### Biosafety.

All of the animal studies were conducted under biosafety level 2 or higher conditions, according to the established guidelines and regulations of the Institutional Animal Care and Use Committee of the Institute of Poultry Research, and all relevant procedures were approved by the committee under license no. 18-02.
